# How Immunotherapy Modified the Therapeutic Scenario of Endometrial Cancer: A Systematic Review

**DOI:** 10.3389/fonc.2022.844801

**Published:** 2022-04-14

**Authors:** Brigida Anna Maiorano, Mauro Francesco Pio Maiorano, Gennaro Cormio, Annamaria Maglione, Domenica Lorusso, Evaristo Maiello

**Affiliations:** ^1^ Oncology Department, Fondazione Casa Sollievo della Sofferenza IRCCS, San Giovanni Rotondo, Italy; ^2^ Department of Translational Medicine and Surgery, Catholic University of the Sacred Heart, Rome, Italy; ^3^ Obstetrics and Gynecology Unit, Department of Biomedical Sciences and Human Oncology, University of Bari “Aldo Moro”, Bari, Italy; ^4^ Obstetrics and Gynecology Department, Fondazione Casa Sollievo della Sofferenza IRCCS, San Giovanni Rotondo, Italy; ^5^ Department of Women and Child Health, Division of Gynaecologic Oncology, Fondazione Policlinico Universitario “A. Gemelli” IRCCS, Rome, Italy; ^6^ Scientific Directorate, Fondazione Policlinico Universitario “A. Gemelli” IRCCS, Rome, Italy

**Keywords:** endometrial cancer (EC), immune checkpoint inhibitors (ICI), immunotherapy, pembrolizumab, lenvatinib, dostarlimab, PD1, microsatellite instability (MSI)

## Abstract

**Background:**

Endometrial cancer (EC) represents the sixth most common female tumor. In the advanced setting, the prognosis is dismal with limited treatment options. Platinum-based chemotherapy represents the actual standard of care in first-line chemotherapy, but no standard second-line chemotherapy is approved, with less than 1/4 of patients responding to second-line chemotherapy. In the last 10 years, immune checkpoint inhibitors (ICIs) have changed the treatment landscape of many solid tumors.

**Methods:**

The review was conducted according to the PRISMA guidelines. We searched EMBASE, MEDLINE, Cochrane Database, and conference abstracts from international societies, up to November 2021. Clinical trials employing ICIs in advanced EC, written in English, were included. Reviews, letters, and commentaries were excluded. The overall response rate (ORR), progression-free survival (PFS), overall survival (OS), and safety (number and grade of treatment-related adverse events [TRAEs]) were evaluated.

**Results:**

15 studies, for a total of 1,627 patients, were included: 14 non-randomized phase I/II trials and 1 randomized phase III trial. Anti-PD1 (pembrolizumab, nivolumab, dostarlimab) and anti-PD-L1 agents (avelumab, atezolizumab, durvalumab) were administered as single agents; pembrolizumab and nivolumab were combined with the tyrosine-kinase inhibitors (TKI) lenvatinib and cabozantinib, respectively; and durvalumab was associated with anti-CTLA4 tremelimumab. 4 studies selected only MSI patients. Single agents determined an ORR from 26.7% to 58% among MSI patients, from 3% to 26.7% among MSS patients. DCR ranged from 53.5% to 88.9% in MSI, 31.4% to 35.2% in MSS patients. The combination of TKI and ICIs determined 32% to 63.6% of ORR in all-comers, 32%–36.2% in MSS patients. 54.2% to 76% of patients developed TRAEs. The combination of ICIs and TKI achieved a higher toxicity rate than single agents (≥G3 TRAEs 88.9%).

**Conclusion:**

ICIs represent an effective option for pretreated advanced EC patients with a tolerable profile. Given the encouraging results in MSI patients, every woman diagnosed with EC should be investigated for MS status. In MSS women, the combination of ICIs and TKI is more effective than monotherapy, notwithstanding safety concerns. PD-L1 cannot predict ICI response, whereas other biomarkers such as MSI and tumor mutational burden seem more accurate. Ongoing randomized trials will further clarify the role of these therapeutic options.

**Systematic Review Registration:**

PROSPERO, CRD42021293538.

## 1 Introduction

With an incidence of approximately 10.8 cases/100,000/year, endometrial cancer (EC) represents the sixth most common cancer among women, accounting for 4.5% of all new cancer diagnoses. The incidence rises with age, being very uncommon before 40 years and reaching 35.2 cases/100,000/year among >50-year-old women, with a median age at diagnosis of 63 years (1–3). Several risk factors for EC have been identified: age, familiar history, previous radiation therapy, obesity, diabetes, metabolic disease, diet, exercise, and general lifestyle (4). Furthermore, menopause, tamoxifen use, birth control pills, intrauterine devices, pregnancy, polycystic ovarian syndrome, and history of endometrial hyperplasia, while affecting circulating sex hormone levels, might contribute to EC development (5). EC represents the 13th cause of cancer-related deaths among women, with a mortality rate of 2.5/100,000/year (1). The 5-year survival rate dramatically drops from 94.9% for localized diseases to 17.8% for the metastatic stage, representing 9% of total diagnoses (2). In the localized setting, surgery is the first-choice treatment, also combined with radiotherapy, whereas chemotherapy represents the cornerstone for the high-risk and advanced diseases. The current standard of care (SOC) for first-line advanced/recurrent EC is the combination of carboplatin and paclitaxel, which guarantees a median overall survival (mOS) of 37 months and a median progression-free survival (mPFS) of 13 months (6). However, there is currently no SOC after platinum progression (7). Response rates (RRs) with single-agent chemotherapy (mainly ifosfamide, docetaxel, doxorubicin, weekly paclitaxel), and endocrine therapy, range from 8% to 24%, with less than 1 year of OS (8, 9). Recent findings have suggested the efficacy of platinum derivatives in “platinum-sensitive” patients (10). However, while platinum rechallenge might be an option in recurrent EC with a long recurrence-free interval, there is clearly a need for new therapeutic options (7, 9, 10).

The Cancer Genome Atlas (TCGA) described at least 4 molecular subtypes of EC: polymerase ϵ (POLE)-mutant, microsatellite instable-high (MSI-H), copy number low, and copy number high. The first two subtypes are associated with a better prognosis. Effectively, up to 30% of EC are MSI-H, characterized by defective proteins that repair DNA through the mismatch repair (MMR) mechanism. MMR-deficient (MMRd) EC accumulates errors in areas of repetitive DNA sequences called microsatellites, developing a high mutational load due to the release of a significant number of neo-antigens, which has been associated with immunotherapy response (11). Indeed, immunotherapy, particularly immune-checkpoint inhibitors (ICIs), represents the current cutting-edge therapy for many solid tumors, including gynecological malignancies (12). It is worthy of note that the Food and Drug Administration (FDA) granted two accelerated approvals of ICIs for pretreated EC patients: pembrolizumab for MSI-H tumors in 2017, and pembrolizumab plus lenvatinib for MS-stable (MSS) disease in 2019 (13, 14). Moreover, in 2021, the European Medial Agency (EMA) approved pembrolizumab and lenvatinib for pretreated EC patients, and dostarlimab for MSI-H EC (15, 16).

We hereby systematically reviewed the clinical trials regarding ICIs for the treatment of advanced EC to evaluate how they might change the clinical approach to this malignancy and future directions for tailored trials. To the best of our knowledge, this is the first systematic review to synthesize the efficacy and safety of clinical trials employing ICIs in EC.

## 2 Materials and Methods

### 2.1 Protocol Registration

We registered the protocol for this systematic review with PROSPERO (CRD42021293538).

### 2.2 Search Strategy and Data Extraction

This systematic review was carried out following the Preferred Reporting Items for Systematic Reviews and Meta-Analysis (PRISMA) statement (17). Two authors (BM and MM) independently performed a literature search of the databases PubMed, EMBASE, and Cochrane Central Register of Controlled Trials, in November 2021. The search terms (“endometrial neoplasms” OR (“endometrial” AND “neoplasms”) OR “endometrial cancer” OR (“endometrial” AND “cancer”) OR “uterine cancer” OR (“uterine” AND “cancer”) AND [“immune checkpoint inhibitors” OR “ICIs” OR “avelumab” OR “nivolumab” OR “atezolizumab” OR “pembrolizumab” OR “durvalumab” OR “tremelimumab” OR “ipilimumab” or “dostarlimab”)] were used. An additional search for conference abstracts from the American Association of Clinical Oncology (ASCO), European Society of Medical Oncology (ESMO), and Society of Gynecologic Oncology (SGO) was also performed. Article citations were manually checked for additional references.

### 2.3 Inclusion and Exclusion Criteria, Population, Intervention, and Outcomes

We included phase I–IV clinical trials reporting efficacy and safety data of ICIs (single agents or combinations) in advanced/recurrent EC patients, written in the English language. From multi-cohort trials, the number and data of EC patients were identified. Meta-analyses, reviews, case reports, correspondences, personal opinions, and *in vitro*/animal studies were excluded. For the selected studies, the following data were collected: trial name, first author, year of publication, phase, number of treated patients, administered drugs and dosage, and primary and secondary endpoints. We specifically addressed the following efficacy outcomes: overall response rate (ORR), disease control rate (DCR), progression-free survival (PFS), and overall survival (OS); for safety, number and grade of treatment-related adverse events (TRAEs).

### 2.4 Risk of Bias

Two reviewers independently assessed the risk of bias. In case of disagreement, a third reviewer was consulted. The Risk Of Bias In Non-randomised Studies - of Interventions (ROBINS-I) tool was used to assess the risk of bias, including eight domains: confounding bias; selection bias; classification bias; deviation from intended interventions bias; missing data; measure outcome bias; selection of the reported results; and overall bias (18).

## 3 Results

**Figure 1:**
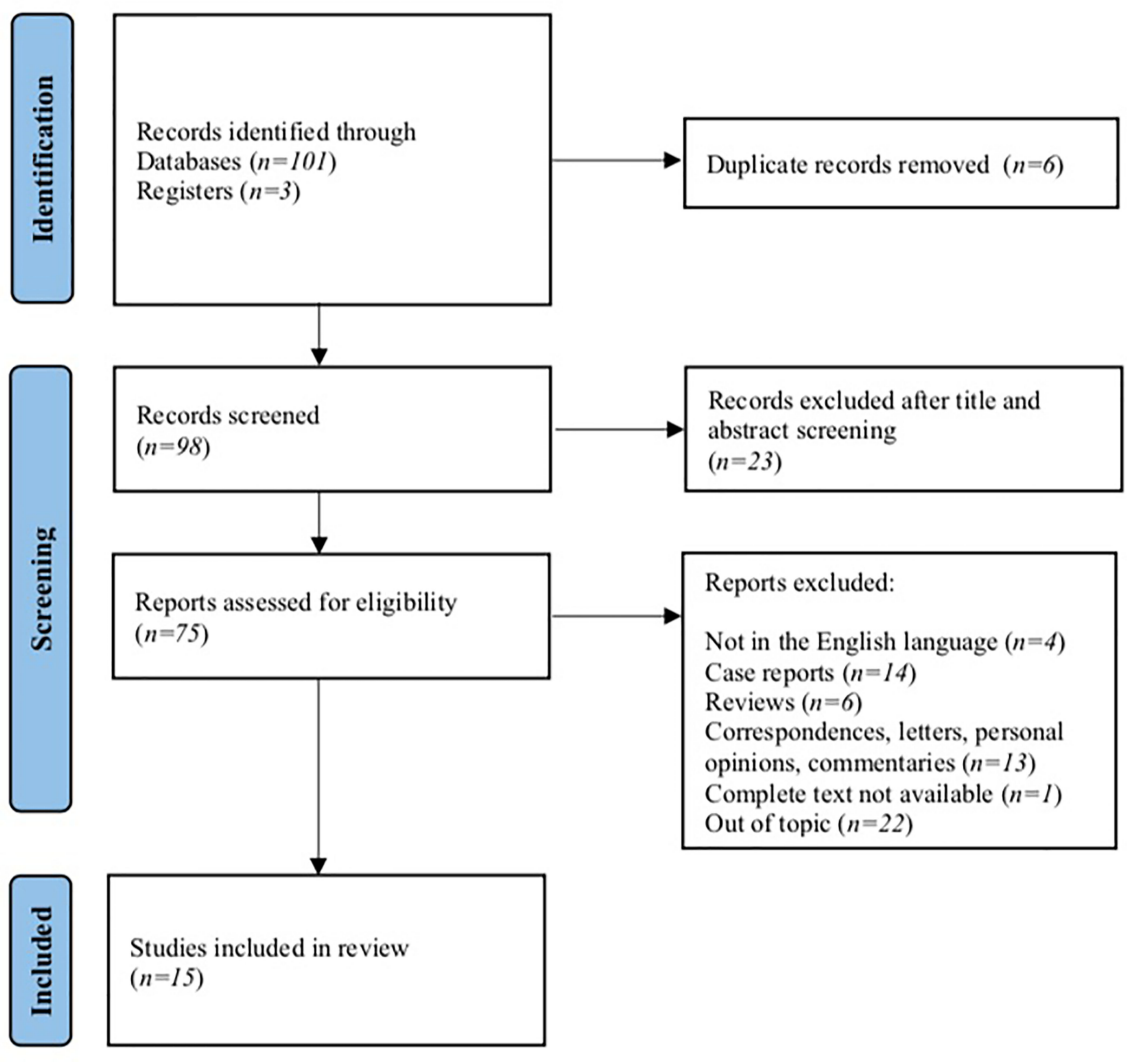
PRISMA flowchart for study selection of the systematic review.

A total of 104 studies were identified from the electronic search. After duplicate removal and title/abstract screening, 75 studies were eligible. After checking inclusion and exclusion criteria, we removed 4 studies for being written in languages other than English, 33 among case reports, reviews, correspondences, personal opinions, or commentaries; in 1 study, the complete text was not available, and 22 reports were removed for focusing on different topics. At the end of the screening, a total of 15 studies were included in our review (**Figure 1**).

### 3.1 Characteristics of the Included Studies

The included studies were 14 phase I/II clinical trials and 1 randomized phase III trial (19–33). No phase IV trial was found. Anti-PD1 agents were used in 11 studies; 4 studies employed anti-PD-L1 agents (in 1 study, anti-CTLA4 was associated). Among PD1, 8 studies used pembrolizumab, 2 nivolumab, and 1 dostarlimab (19–29). Anti-PD-L1 agents consisted of avelumab (1 study), atezolizumab (1 study), and durvalumab (2 studies) (30–33). Anti-PD1 was administered as a single agent in 7 studies (19–23, 27, 29). Pembrolizumab was combined with the tyrosine-kinase inhibitor (TKI) lenvatinib in 3 studies, nivolumab with the TKI cabozantinib in 1 study (24–26, 28). Anti-PD-L1 was administered as monotherapy in 3 studies (30–32). Durvalumab was associated with anti-CTLA-4 tremelimumab in 1 study (33). No study of single-agent anti-CTLA-4 was found. Pembrolizumab was administered every 3 weeks (q3w) at the fixed dosage of 200 mg in 5 studies, 10 mg/kg in 3 studies; nivolumab was administered at the flat dose of 240 mg every 2 weeks (q2w), and in 1 study the shift to 480 mg every 4 weeks (q4w) was planned; atezolizumab was administered either at 1,200 mg or at 15 mg/kg q3w; the avelumab dosage was 10 mg/kg q2w, and dostarlimab was started at the dosage of 500 mg q3w then continued at 1,500 mg every 6 weeks (q6w); and durvalumab was administered at the fixed dosage of 1,500 mg q4w alone or combined with tremelimumab 75 mg q4w. 1,627 patients were treated, ranging from 9 to 827. The overall response rate (ORR) was the most frequent primary endpoint (11 studies): it was defined as the percentage of patients achieving a complete response (CR) or a partial response (PR) (19–24, 26, 27, 29, 30, 33). In one study, the primary endpoint was defined as objective tumor response rate (OTRR—defined as the sum of complete and partial responses divided for the total number of patients) (32). Progression-free survival (PFS)—defined as the time from randomization to disease progression or death, whichever occurred first—was the primary endpoint in 4 studies (20, 25, 28, 30). Overall survival (OS)—defined as the time from randomization to death—was assessed as a co-primary endpoint with PFS in the only selected phase III study (25). PFS, OS, duration of response (DOR), and safety were most commonly investigated among secondary endpoints. Quality of life was addressed as the secondary endpoint only in one study (25). **Table 1** shows the main characteristics of the included studies. No serious risk of bias emerged (**Figure 1**, **Supplementary Material**).

**Table 1:** Trials of ICIs in advanced/recurrent EC.

Author	Study name	Phase	Target population *(number of pts)*	Administered drugs	Primary EP	Secondary EP	Results				
							ORR	DCR	PFS	OS	Safety
Marabelle et al. (19)	KEYNOTE-158 (NCT02628067)—cohorts D/K	II	MSI EC (n = 79)	Pembrolizumab 200 mg q3w	ORR	DOR, PFS, OS	ORR 48% (95% CI, 36.7–59.6%)	DCR 83.5%	mPFS 13.1 mos (95% CI, 4.3–34.4 mos)	mOS NR (95% CI, 27.2 mos-NR)	TRAEs 76%, no G5
Le et al. (20)	NCT01876511—cohort C	II	MSI EC (n = 15)	Pembrolizumab 10 mg/kg q2w	20w-irPFS, 28-mos ORR, 20w-PFS	4y-OS, 28w-irPFS, 28w-PFS, 28-mos DCR	ORR 55%	DCR 73.3%	20w-irPFS 67%	mOS 148.8 wks (94.7-NA)	≥G3 TRAEs 27.7%
									20w-PFS 68% (56-83)		
Fader et al. (21)	NA	II	MSI EC of endometrioid histology (n = 9)	Pembrolizumab 10 mg/kg q2w	ORR	NA	ORR 56% (95% CI, 21–86%)	DCR 88.9%	NA	mOS NR (12 mos OS 89%)	No ≥G3 TRAEs
Roque et al. (22)	NCT02899793	II	MSI-H EC with Lynch syndrome (n = 6) or sporadic MLH1 mutations (n = 18)	Pembrolizumab 200 mg q3w	ORR, safety	PFS, OS	ORR 58% (95% CI, 36.6–77.9%)	NA	3-yr PFS 30% (sporadic) (p = 0.017)	3-yr OS 100% (Lynch), 43% (sporadic) (p = 0.043)	≥G3 TRAEs 6.8%
							ORR 100% (Lynch) vs. 44% (sporadic) (p = 0.024)				
Ott et al. (23)	KEYNOTE-028 (NCT02054806)	Ib	PD-L1^+^ EC (n = 23)	Pembrolizumab 10 mg/kg q2w	ORR	DOR, PFS, OS, safety	ORR 13% (95% CI, 2.8% to 33.6%)	DCR 26.1%	mPFS 1.8 mos (95% CI, 1.6–2.7 mos)	mOS NR (95% CI, 4.3 mos-NR)	TRAEs 54.2%, G3 16.7%, no G4
Makker et al. (24)	KEYNOTE-146/Study 111 (NCT02501096)	Ib/II	EC (n = 108) Stratification: MSI (n = 11) MSS (n = 94)	Pembrolizumab 200mg q3w + lenvatinib 20 mg daily	ORR24w	DOR, PFS, OS	ORR24w 38.0% (95% CI, 28.8%–47.8%)	DCR 84.7% (95% CI, 77.1%–90.5%)	mPFS 7.4 mos (95% CI, 5.3–8.7 mos)	mOS 16.7 mos (95% CI,15.0 mos-NR)	≥G3 TRAEs 69.4%
							MSI subgroup: ORR24w 63.6% (95% CI, 30.8%–89.1%)	MSI subgroup: DCR 90.9% (95% CI, 58.7%–99.8%)	MSI subgroup: mPFS 18.9 mos (95% CI, 4-NR)	MSI subgroup: NR	
							MSS subgroup: ORR24w 36.2% (95% CI, 26.5-46.7%)	MSS subgroup: DCR 84% (95% CI, 75%-90.8%)	MSS subgroup: mPFS 7.4 mos (95% CI, 5-8.7 mos)	MSS subgroup: mOS 16.7 mos (95% CI, 15–NR)	
Makker et al. (25)	KEYNOTE-775/Study 309 (NCT03517449)	III	EC (n = 827), randomized: Exp: n = 411	Pembrolizumab 200 mg q3w plus lenvatinib 20 mg daily or TPC (doxorubicin 60 mg/m^2^ q3w or paclitaxel 80 mg/m2, 3 weeks on, 1 week off)	PFS OS	ORR, safety, QoL	MSS subgroup: ORR 30% (95% CI, 26%–36%) vs. 15% (95% CI, 12%–19%, p<0.0001)	NA	MSS subgroup: mPFS 6.6 mos (95% CI, 5.6–7.4 mos) vs. 3.8 mos (95% CI, 3.6–5.0 mos); HR 0.6 (95% CI, 0.50–0.72; p < 0.0001)	MSS subgroup: mOS 17.4 mos (95% CI, 14.2–19.9 mos) vs. 12 mos (95% CI, 10.8–13.3 mos); HR 0.68 (95% CI, 0.56–0.84; p = 0.0001)	≥G3 TRAEs 88.9% (P+L arm) and 72.7% (CTX arm); combo arm: 30.8% discontinued pembrolizumab, 18.7% discontinued lenvatinib, 14% discontinued both pembro and lenvatinib
			Ctrl: n = 416								
			MSS: n = 697								
			MSI: n = 130				All-comers: ORR 31.9% vs. 14.7%		All-comers: mPFS 7.2 vs. 3.8 mos; HR 0.56	All-comers: mOS 18.3 vs. 11.4 months; HR 0.62	
Taylor et al. (26)	NCT02501096—EC cohort	Ib/II	EC - not selected for biomarkers (n = 23)	Pembrolizumab 200 mg q3w plus lenvatinib 20 mg daily	ORR24w	ORR, PFS, DOR, DCR	ORR24w and overall ORR 52% (95% CI, 30.6%–73.2%)	DCR 95.6%	mPFS 9.7 mos (95% CI, 4.2 mos-NR)	NA	TRAEs 97%, ≥G3 TRAEs 67%, 2 TR-deaths
Tamura et al. (27)	JapicCTI-163212	II	EC—not selected for biomarkers (n = 22)	Nivolumab 240 mg q2w	ORR	OS, PFS, DCR, safety	ORR 23% (95% CI, 11%–38%)	DCR 68.2%	mPFS 3.4 mos (95% CI, 2.0–5.4 mos)	NA	TRAEs 61%, ≥G3 TRAEs 17%
Lheureux et al. (28)	NCT03367741	II	EC—not selected for biomarkers, randomized (n = 76)	Arm A: nivolumab 240 mg q2w (480 mg q4w after 4 cycles) + cabozantinib 40 mg daily	PFS	OS, ORR, safety	ORR 25% (Arm A), 16.7% (Arm B)	DCR 69.4% (Arm A), 27.8% (Arm B)	Arm A: mPFS 5.3 mos (95% CI, 3.5–9.5 mos)	NA	Most common AEs (>G1/G2): diarrhea (47.2%), hypertransaminasemia (44.4%), fatigue (38.9%), nausea (30.6%)
			Arm A: n = 36 Arm B: n = 18 Exploratory Arm C (carcinosarcoma or EC progressive to immunotherapy): n = 29	Arm B: nivolumab 240 mg Arm C: nivolumab + cabozantinib					Arm B: mPFS 1.9 mos (95% CI, 1.6–3.8 mos)		
Oaknin et al. (29)	GARNET (NCT02715284)	I/II	103 MSI EC, 142 MSS EC	Dostarlimab 500 mg q3w x 4 → 1,000 mg q6w	ORR	DOR, DCR	MSI: ORR 44.7% (95% CI, 34.9%–54.8%)	MSI: DCR 57.3%	MSI: mPFS 8.1 months (95% CI, 3.0–18.0 months)	mOS NR	TRAEs: 63.5% (MSI), 71.7% (MSS); serious TRAEs: 13.5% (MSI), 19.3% (MSS)
							MSS: ORR 13.4% (95% CI, 8.3%–20.1%)	MSS: DCR 35.2%			
Kostantinopoulos et al. (30)	NCT02912572	II	MSI/*POLE* mutated cohort (n = 15)	Avelumab 10 mg/kg q2w	PFS6, ORR	PFS, OS, safety	MSI cohort: ORR 26.7% (95% CI, 7.8%–55.1%)	DCR 53.3%	MSI cohort: PFS6 40% (95% CI, 16.3%–66.7%)	MSI cohort: mOS NR	TRAEs 71%, G3 TRAEs 19.4%
			MSS cohort (n = 16) → closed for futility								
Fleming et al. (31)	NCT01375842	Ia	PD-L1^+^ EC, then amended to all patients (n = 15)	Atezolizumab 1,200 mg or 15 mg/kg q3w	Safety, clinical activity	NA	ORR 13%	DCR 26.7%	mPFS 1.7 mos (95% CI, 0.6–11 mos)	mOS 9.6 mos (95% CI, 0.6–11.8 mos)	47% TRAEs, no G4-5 TRAEs
Antill et al. (32)	PHAEDRA (ANZGOG1601/ACTRN12617000106336)	II	EC (n = 71): 36 MSI 35 MSS	Durvalumab 1,500 mg q4w	OTRR (iRECIST)	PFS, OS	MSI: OTRR 47% (95% CI 32%–63%)	MSI: DCR 63.9%	MSI: mPFS 8.3 mos	MSI: mOS NR 12-mos OS 71%	NA
							MSS: OTRR 3% (95% CI 1%–15%)	MSS: DCR 31.4%	MSS: mPFS 1.8 mos	MSS: mOS 11.5 mos 12-mos OS 51%	
Rubinstein et al. (33)	NCT03015129	II	EC (>10 MSI or carcinosarcoma per arm) (n = 54; D: n = 27, D+T: n = 27)	Durvalumab 1,500 mg q4w or plus tremelimumab 75 mg q4w → durvalumab 1,500 mg q4w	ORR	NA	D arm: ORR 14.8% (90% CI, 6.6%–100%)	NA	D arm: mPFS 7.6w PFS24w 13.3% (95% CI, 6.1%–100%)	NA	D arm: 7% G3, 4% G4 TRAEs
							DT arm: ORR 11.1% (90% CI, 4.2%–100%)		DT arm: mPFS 8.1w PFS24w 18.5% (95% CI, 10.1%–100%)		DT arm: 32% G3, 11% G4 TRAEs

AEs, adverse events; CI, confidence interval; CR, complete response; CTX, chemotherapy; DCR, disease control rate; DOR, duration of response; EC, endometrial cancer; HR, hazard ratio; irORR, immune-related objective response rate; irPFS, immune-related progression free survival; mDOR, median duration of response; mOS, median overall survival; mPFS, median progression free survival; MSI, microsatellite instability; MSS, microsatellite stability; NA, not available; NR, not reached; ORR, objective response rate; ORR24w, objective response rate at 24 weeks; OS, overall survival; OTRR, objective tumor response rate; PD-L1, programmed death-ligand 1; PFS, progression free survival; PFS24w, progression free survival at 24 weeks; PFS6, progression-free survival at 6 months; POLE, polymerase epsilon; PR, partial response; QoL, quality of life; SD, stable disease; TPC, treatment of physician’s choice; TRAE, treatment-related adverse event.

**Figure 2:**
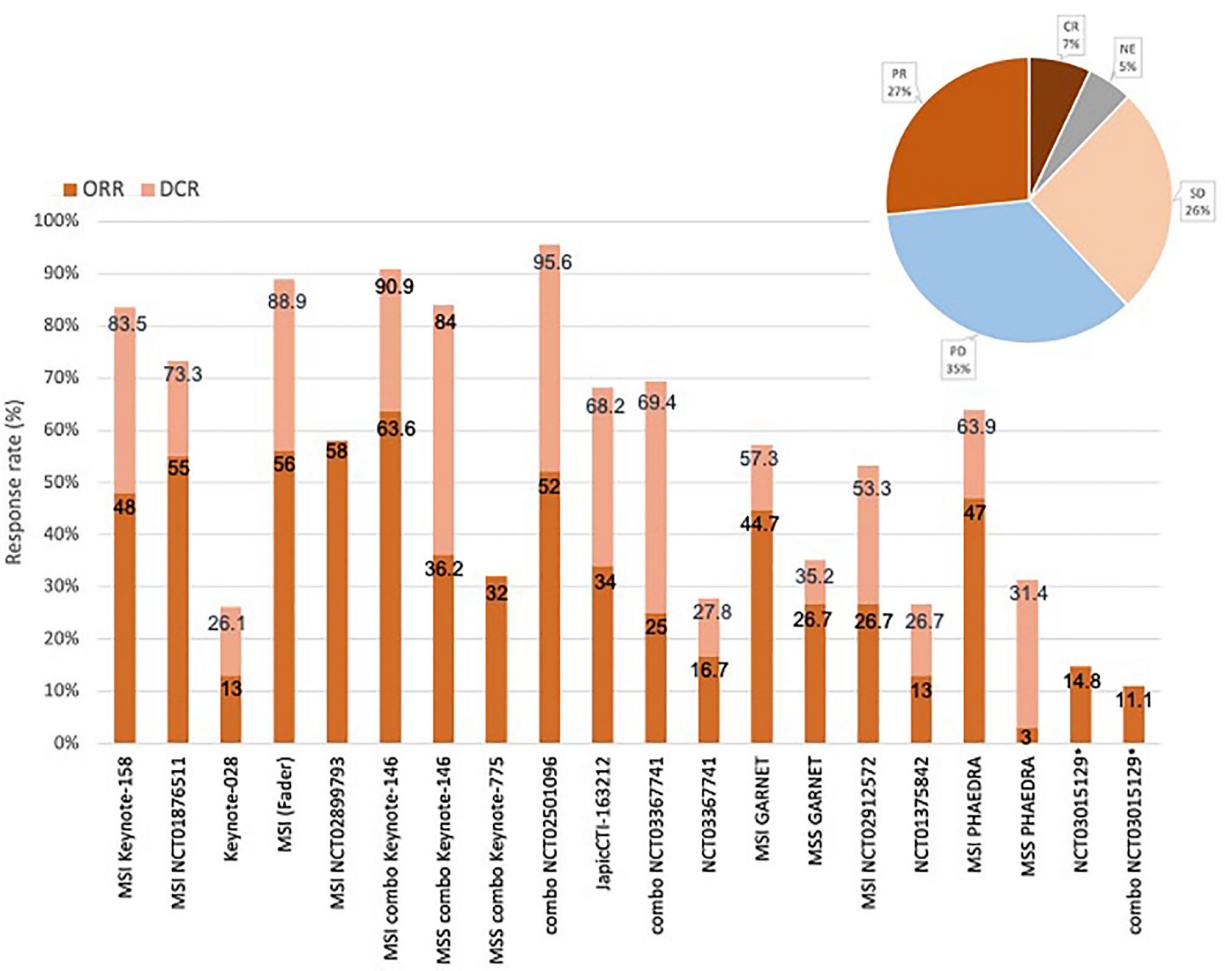
Responses and response rates of the included studies. Overall response rate (ORR) ranged from 3% to 58%. ORR to single agents ranged from 26.7% to 58% for MSI patients, 3% to 26.7% for MSS patients (studies that selected MSI and MSS patients are indicated in the figure). In the combination ICIs-TKI studies (“combo” in the figure), ORR was 32% to 52%, reaching 36.2% in MSS patients, 63.6% in MSI patients. DCR ranged from 26.1% to 95.6% in all studies, with peaks in MSI patients (around 90% as single agents or combinations) and MSS patients in case of combination (84%). Objective tumor response rate (OTRR—marked with *) to the combo durvalumab plus tremelimumab was 11.1%. Types of responses recorded in the studies were: 35% progressive disease (PD), 27% partial response (PR), 26% stable disease (SD), 7% complete response (CR).

Overall, ORR ranged from 3% to 63.6%. When considering only MSI patients, ORR to single agents ranged from 26.7% to 58% (median 48%), while when including only MSS patients, ORR was 3% to 26.7% (median 14.8%). In the combination ICI-TKI studies, ORR was 32% to 63.6%; KEYNOTE-146 reported an ORR of 63.6% in MSI patients (n = 11), whereas MSS patients reached an ORR of 32% to 36.2% in KEYNOTE-146 and -775. 12 studies reported DCR, ranging from 26.1% to 95.6% in all-comers, with peaks in MSI patients treated with single agents (53.5% to 88.9%), and patients receiving the combination of pembrolizumab/lenvatinib (90.9% in MSI, 84% in MSS patients). No additional benefit derived from the dual-ICI combination, as durvalumab plus tremelimumab determined an ORR of 11.1%. Considering the types of responses, 35% of patients developed a PD, and 27% PR, 26% SD, and 7% of CR were observed (**Figure 2**).

**Figure 3:**
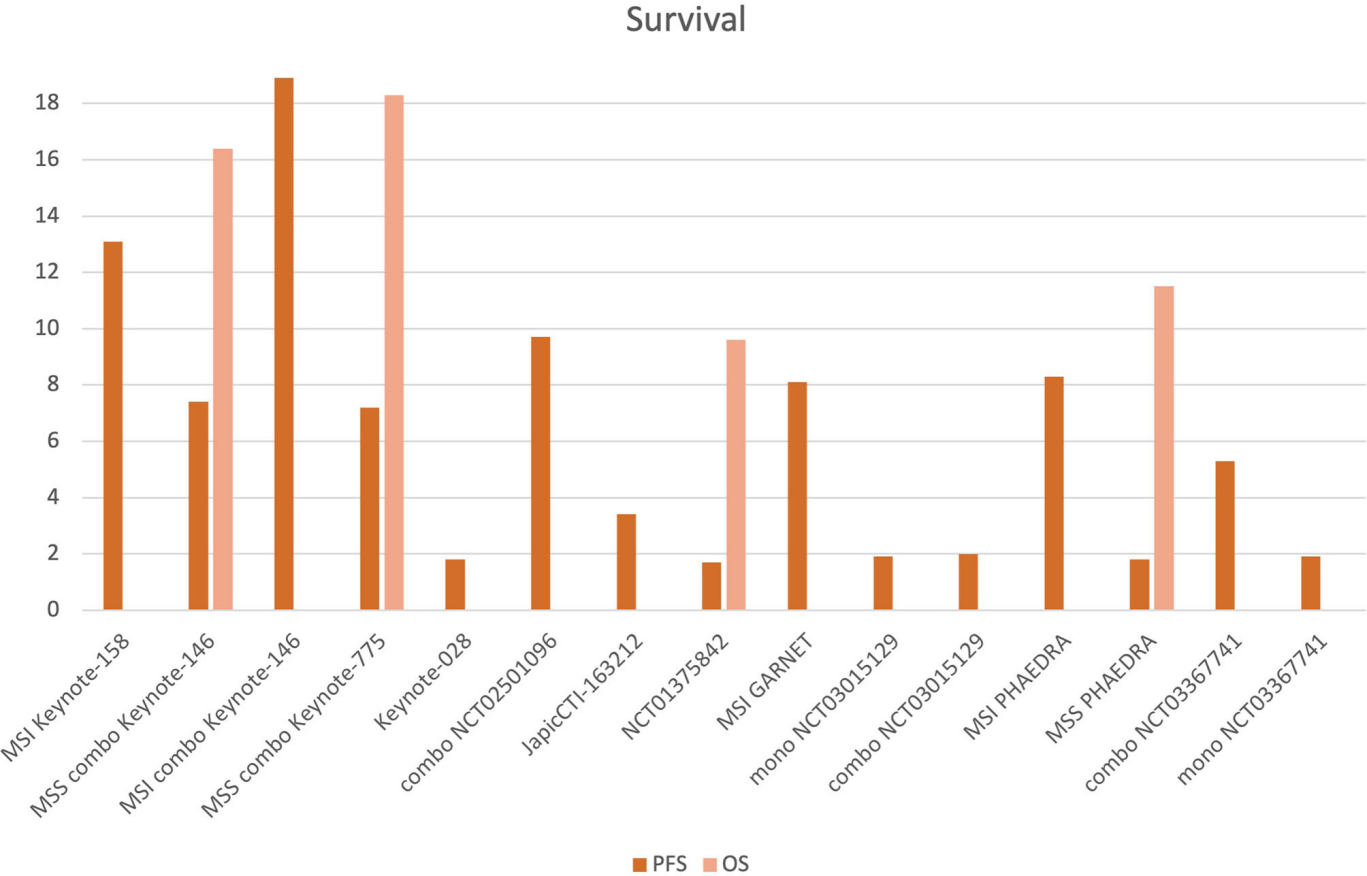
Progression-free survival (PFS) and overall survival (OS) of the included studies. 11 studies reported mPFS, which ranged from 1.7 to 18.9 months. Among MSI patients treated with ICI monotherapy, mPFS was 8.1 months (5.6–13.1 mos). With combination ICIs-TKI, mPFS ranged from 7.2 to 18.9 mos (among the 11 MSI patients of Keynote-146), ranging from 6.6 to 7.4 months for MSS patients. mOS was available for 5 studies, ranging from 9.6 to 18.3 mos (MSI, MSS groups and combination—”combo” studies are indicated.

11 studies reported mPFS that ranged from 1.7 to 18.9 months. Among MSI groups, mPFS to ICI monotherapy was 8.1 months (range 5.5–13.1 mos). With combination ICI–TKI, mPFS ranged from 7.2 to 8.7 mos in all-comers, with similar benefits in MSS patients (6.6–7.4 months), reaching 18.9 months among 11 MSI patients in KEYNOTE-146. mOS was available only for 5 studies, ranging from 9.6 to 18.3 months (**Figure 3**).

With ICI monotherapy, 54.2% to 76% of patients developed TRAEs, of which 6.8% to 27.7% were ≥G3. The combination of ICIs and TKI achieved a higher toxicity rate than single agents (≥G3 TRAEs 67%–88.9%). The dual anti-PD1/anti-CTLA4 inhibition determined 44% of serious TRAEs (**Table 1**).

### 3.2 ICIs Targeting PD1

#### 3.2.1 Pembrolizumab

Pembrolizumab monotherapy was investigated in 5 trials, 1 of which was a phase Ib study and 4 were phase II studies, for a total of 150 patients (range 9–79) (19–23). Pembrolizumab was administered at the dosage of 10 mg/kg q3w in all studies except for KEYNOTE-158 and NCT02899793, which used the flat dose of 200 mg q3w. Patients were selected for MSI-H/MMRd status in 4 studies (19–22). Among them, NCT02899793 was a pilot study comparing MSI-H patients with Lynch syndrome versus sporadic MLH1 mutations (22). In KEYNOTE-028, patients were included in case of PD-L1 positivity (cutoff 1% on tumor or inflammatory cells or in the stroma) (23).

Cohorts D/K of the KEYNOTE-158 phase II trial (NCT02628067) included 79 MSI-H EC patients. The primary endpoint was ORR; DOR, PFS, and OS were secondary endpoints. ORR was 48% (95% confidence interval [CI], 36.7%–59.6%), DCR 83.5%. mPFS was 13.1 months (95% CI, 4.3–34.4 mos), mOS not reached (NR; 95% CI, 27.2 mos-NR). 14 CR were recorded (of note, EC recorded one of the highest CR rates among the solid tumors included in the study). TRAEs were reported in 76% of patients, with no grade 5 events (19). Similar results were achieved in 15 MMRd and 9 MSI-H EC patients in two phase II trials, with—primary endpoint—ORRs of 55% and 56% and DCRs 73.3% and 88.9%, respectively. In the first study, mOS was 148.8 weeks. ≥G3 toxicities were reported in 27.7% of MMRd patients, but no ≥G3 TRAE was recorded among 9 MSI-H patients (20, 21). In the pilot phase II NCT02899793 study, 24 MSI-H EC patients were treated with pembrolizumab, reaching an ORR of 58% (95% CI, 36.6%–77.9%), which was 100% in patients with Lynch syndrome, versus 44% of sporadic cases (p = 0.024). Patients with Lynch syndrome (n = 6) were all alive after 3 years, whereas in sporadic patients (n = 18), 3-year PFS and 3-year OS were 30% (p = 0.017) and 43% (p = 0.043), respectively. 6.8% ≥G3 TRAEs were recorded (22). In the multi-cohort phase Ib KEYNOTE-028 study (NCT02054806), patients with locally advanced or metastatic PD-L1-positive solid tumors were enrolled. ORR was the primary endpoint; secondary endpoints included safety, DOR, PFS, and OS. 23 EC patients were included in the efficacy analysis, with an ORR of 13.0% (95% CI, 2.8%–33.6%), DCR of 26.1%, mPFS of 1.8 months (95% CI, 1.6–2.7 mos), and mOS NR. Among these patients, only one was POLE-mutant and one MSI-H; the remaining were stable or not evaluable for MSI. The only POLE-mutant patients achieved a PR, and the MSI patient had PD; among all patients, 3 PR, 3 SD, and 13 PD were recorded. 13 TRAEs and 4 G3 TRAEs, but no G4 AEs, occurred (23).

The combination of pembrolizumab 200 mg q3w and lenvatinib 20 mg daily was evaluated in 3 studies enrolling pretreated EC patients (24–26). There were 2 phase Ib/II trials investigating ORR (24 weeks after treatment starting) as the primary endpoint, and a phase III trial with PFS and OS as co-primary endpoints. Overall, 542 patients received the combination. In the phase Ib/II KEYNOTE-146/Study111 (NCT02501906), 108 patients were included. The study demonstrated a 24-week ORR (primary endpoint) of 38.0% (95% CI, 28.8%–47.8%), ranging from 36.2% (26.5%–46.7%) in patients with MSS tumors (n = 94) to 63.6% (95% CI, 30.8%–89.1%) in patients with MSI-H tumors (n = 11). DCR was 84.7% (95% CI, 77.1%–90.5%) in all-comers, 84% (95% CI, 75%–90.8%) in MSS patients, and 90.9% (95% CI, 58.7%–99.8%) in MSI patients. mDOR was 21.2 months. mPFS was similar between all-comers (7.4 months; 95% CI, 5.3–8.7 mos) and MSS patients (7.4 mos; 95% CI, 5.0–7.6 mos) and reached 18.9 months in MSI patients (95% CI, 4 mos-NR). mOS was 16.7 months in all patients (95% CI, 15 mos-NR), 16.4 months in MSS (95% CI, 13.5-25.9 mos), and NR in MSI patients. 69.4% of women experienced ≥G3 TRAEs, most frequently hypothyroidism (47.6%). 6 treatment-related deaths were reported (24). This study led the FDA to grant breakthrough therapy designation to the combination of pembrolizumab and lenvatinib for pretreated MSS EC women (14). The KEYNOTE-775/Study309 (NCT03517449) is the phase III confirmatory trial for KEYNOTE-146. 827 women were randomized to receive lenvatinib 20 mg plus pembrolizumab 200 mg q3w (n = 411) versus treatment of physician choice (doxorubicin or weekly paclitaxel) (n = 416). PFS and OS were the co-primary endpoints, while ORR, safety, and quality of life were secondary endpoints. In all-comers, ORR was 32% vs. 15%, mPFS was 7.2 vs. 3.8 mos (HR 0.56), and mOS was 18.3 vs. 11.4 months (HR 0.62), respectively. In the MSS cohort, ORR was 30% vs. 15%, mPFS was 6.6 vs. 3.8 mos (HR 0.6; 95% CI, 0.50–0.72; p < 0.0001), and mOS was 17.4 vs. 12 mos (HR 0.68; 95% CI, 0.56–0.84; p = 0.0001), respectively. ≥G3 TRAEs were observed in almost 89% of the lenvatinib/pembrolizumab arm (most commonly hypertension) and 72.7% of the chemotherapy arm, with 30.8% discontinuing pembrolizumab, 18.7% discontinuing lenvatinib, and 14% discontinuing both pembro and lenvatinib in the combination arm (25). 23 EC patients were treated in the phase Ib/II NCT02501096 trial. The—primary endpoint—24-week ORR was 52% (95% CI, 30.6%–73.2%), with 2 CR and 10 PR, and DCR was 95.6%. mPFS was 9.7 months (95% CI, 4.2 mos-NR). ≥G3 TRAEs occurred in 67% of patients, hypertension, fatigue (12%), diarrhea (9%), proteinuria (8%), and increased lipase levels (7%) being the most common. 2 treatment-related deaths were recorded (26).

#### 3.2.2 Nivolumab

As monotherapy, nivolumab was tested in the JapicCTI-163212 phase II trial on the Japanese population. 22 patients in the EC cohort received nivolumab 240 mg q2w, with ORR as the primary endpoint, and OS, PFS, DOR, and safety as secondary endpoints. ORR was 23% (95% CI, 11%–38%), mPFS 3.4 months (95% CI, 2.0–5.4 mos). DCR was 68.2%, with no CR observed. 61% of patients developed a TRAE, which was >G3 in 17% of cases. Exploratory biomarker analysis included PD-L1^+^ and MSI-H patients: similar ORRs were recorded between PD-L1-positive and -negative patients; none of the 6 MSS-stable patients and both 2 MSI women responded to nivolumab (27).

Nivolumab was tested in combination with cabozantinib in the randomized three-cohort phase II NCT03367741 trial. Patients were eligible if they had recurrent EC progressing to at least 1 platinum-based chemotherapy in the first two cohorts; a third exploratory cohort included patients with carcinosarcoma or progressive patients to previous immunotherapy. 76 women were randomized to receive nivolumab (240 mg q2w for the first 4 cycles, followed by 480 mg q4w) plus cabozantinib 40 mg daily (arm A; n = 36) or nivolumab monotherapy (arm B; n = 18); the exploratory cohort (cohort C) of 9 carcinosarcoma and 20 ICI-progressing EC patients received nivolumab plus cabozantinib. PFS was the primary endpoint. OS, ORR, and safety were assessed as secondary endpoints. mPFS was 5.3 months (95% CI, 3.5–9.5 mos) in arm A, and 1.9 months (95% CI, 1.6–3.8 mos) in arm B. ORR was 25% and 16.7% in arms A and B, respectively. DCR was 69.4% in arm A, 27.8% in arm B. Diarrhea (47.2%), transaminase increase (44.4%), fatigue (38.9%), and nausea (30.6%) were the most common TRAEs. In the exploratory cohort, among 9 patients with carcinosarcoma, 1 PR and 4 SD were observed; among the 20 immunotherapy-progressive EC women, 6 responses and 8 SD were recorded (28).

#### 3.2.3 Dostarlimab

In the phase I/II GARNET study, 103 women with MSI and 142 with MSS advanced/recurrent EC received 500 mg of dostarlimab q3w for 4 doses and then 1,000 mg q6w. The primary endpoint was ORR; DCR and DOR were secondary endpoints. Dostarlimab showed a meaningful clinical benefit, with an ORR of 44.7% (95% CI, 34.9%–54.8%) in MSI, and 13.4% (95% CI, 8.3%–20.1%) in MSS women. DCR was 57.3% and 35.2% in the MSI and MSS groups, respectively. 63.5% MSI and 71.7% MSS patients developed TRAEs, of which 13.5% and 19.3% were serious AEs (29).

### 3.3 ICIs Targeting PD-L1

#### 3.3.1 Avelumab

In a single-arm phase II study (NCT02912572), two cohorts of EC patients (15 MSI and 16 MSS) were treated with avelumab 10 mg/kg q2w until progression or unacceptable toxicity. The co-primary endpoints were the frequency of patients with a PFS of at least 6 months after initiating therapy (PFS6), and ORR. Secondary endpoints were PFS, OS, and safety. The MSS cohort was closed after meeting futility criteria, whereas MSI patients exhibited an ORR of 26.7% (95% CI, 7.8%–55.1%) and a PFS6 rate of 40% (95% CI, 16.3%–66.7%), regardless of PD-L1 status. DCR was 53.3%. 71% of patients developed a TRAE, of which 19.4% were G3 TRAEs (30).

#### 3.3.2 Atezolizumab

Atezolizumab 1,200 mg or 15 mg/kg q3w was administered during a phase Ia study (NCT01375842) to 15 patients with advanced/recurrent EC, 93% of which progressed to ≥2 prior systemic therapies. 7/15 patients were MSS, 1 was MSI-H, and 7 had MS-unknown status. Patients were initially evaluated for PD-L1 status (with a cutoff for positivity of 5%); the study was then extended to all patients independently from PD-L1. Atezolizumab clinical benefit seemed to be highly related to PD-L1 expression and MSI. ORR was 13%, DCR 26.7%; 2 PR were observed, 1 in a MSI patient, 1 in a patient with 70% of TIL infiltration, both of which were PD-L1 positive. mPFS was 1.7 months (95% CI, 0.6–11 mos), mOS 9.6 months (95% CI, 0.6–11.8 mos). 47% of patients developed a TRAE, but no G4-G5 events were recorded (31).

#### 3.3.3 Durvalumab

In the phase II PHAEDRA (ANZGOG1601) trial, durvalumab 1,500 mg q4w was administered to 71 patients with MSS (n = 35) or MSI (n = 36) advanced EC. The objective tumor response (OTR, including CR and PR by RECIST criteria) rate was the primary endpoint while PFS and OS were secondary endpoints. Among MSI, the OTR rate was 47% (95% CI, 32%–63%), with 6 CR and 11 PR, and DCR was 63.9%. mPFS was 8.3 mos, and mOS was NR with a 12-mos OS rate of 71%. In the MSS subgroup, the OTR rate was 3% (95% CI, 1%–15%), DCR was 31.4%, with only 1 PR and 10 SD observed, and mPFS was 1.8 mos, mOS 11.5 mos, and 12-mos with an OS rate of 51% (32). In the NCT03015129 phase II trial, EC patients were randomized to receive durvalumab 1,500 mg q4w with or without tremelimumab 75 mg q4w for 4 cycles, followed by durvalumab maintenance, until progression or unacceptable toxicity. ORR was the primary endpoint. At least 10 patients with carcinosarcoma or MSI per arm were planned: as 2 patients were excluded due to early death, 27 patients per arm were considered. 5 patients were MSI, 48 MSS; in 3 cases, the MS status was unknown. In the single-agent arm, there were 1 CR (MSS) and 3 PR (2 MSS and 1 MSI), reaching an ORR of 14.8% (90% CI, 6.6%–100%). mPFS was 7.6 weeks, PFS24wks was 13.3% (90% CI, 6.1%–100%), and mDOR was 16 wks. Regarding the combination arm, 2 CR (1 MSI, 1 MSS) and 1 PR (MSS) were found. ORR was 11.1% (90% CI, 4.2%–100%), mPFS was 8.1 wks, and PFS24wks was 18.5% (90% CI, 10.1%–100%). As for safety, G3 TRAEs occurred in 7% of patients in the single-agent arm and 32% of patients in the double-agent arm, with fatigue and diarrhea as the most common TRAEs. G4 TRAEs occurred in 4% of single-agents and 11% of combination groups (33).

## 4 Discussion

EC profoundly impacts women’s health in terms of morbidity and mortality, and dismal results are reported in platinum-progressing patients (1–3, 7). Therefore, the search for effective treatments beyond the first line represents one of the most important unmet needs for this malignancy (7). In the last 10 years, ICIs have brought a paradigm shift in the therapy of many solid tumors. Effectively, EC represents a unicum among gynecological tumors, as ICI approvals have already occurred in pretreated patients (13–16). The results of our systematic review confirm that ICIs are effective in patients with pretreated advanced EC. ORR ranges from 3% to 63.6%, DCR ranges from 26.1% to 95.6%. Overall, response to ICIs is tripartite: 1/3 of progressing patients, 1/3 of responding patients (CR+PR), and 1/3 of disease stability. Therefore, 2 out of 3 women might benefit from ICIs. The impressive results of the KEYNOTE-158 (cohorts D/K) and GARNET trials justify the use of anti-PD-1 in MSI-H tumors, confirming the FDA and EMA approvals (19, 29). The results of the other trials corroborate the efficacy of single-agent ICIs in MSI-H patients, as ORR ranges from 26.7% to 58% (19–22, 29, 30, 32). The efficacy is far more limited in MSS patients, with ORRs ranging from 3% to 26.7% (29, 32). However, another effective approved therapy for those patients is represented by the double association of pembrolizumab and the TKI lenvatinib, with ORRs of 32%–36.2% (24–26, 28). Besides ORR, the amount of disease stability is considerable, with DCRs from 26.1% to 95.6%, in line with the effect of ICIs: effectively, since ICIs restore a tumor-specific immune response, novel patterns of response are observed after immunotherapy that differ from chemotherapy and target therapies, such as durable responses that not always start rapidly but can persist even after ICI interruption (34). OS results are incomplete for most studies, however—as previously described in other solid tumors, the ICI effect is prolonged and OS is improved beyond PFS. This is in line with studies conducted in other solid tumors, as—once established—the immune response persists in the long run (**Figure 2**).

Despite these premises, we should point out that at least 1 out of 3 EC patients progresses to ICIs. Therefore, the search for predictive biomarkers is of utmost importance for better patient selection and treatment strategy definition. Regarding PD1 and PD-L1, EC shows the highest rates of expression among gynecological tumors, with PD1 positivity reported in around 75% of cases, and PD-L1 positivity ranging from 25% to 100% of EC specimens (especially in the endometrioid subtype), associated with advanced stages and poor prognosis (35). Controversial data regard the correlation between PD-L1 expression and MS status, with evidence of higher PD-L1 levels in MSI than MSS EC in some cases, but no differences in other reports (36–38). Moreover, results regarding the predictive role of PD-L1 for ICIs are inconsistent (23, 27). Differently from PD-L1, tumor mutational burden (TMB) seems useful for identifying a subgroup of patients who could better respond to ICIs (39, 40). In a biomarker analysis of KEYNOTE-158, 790 patients were evaluable for TMB: 102 patients (13%) were TMB high (having >10 mutations per megabase) and reached an ORR of 29% versus 6% of the non-TMB-high group (40). We should deepen the predictive role of TMB in EC, especially POLE-mutant and MSI tumors, which are associated with high TMB (39). A high number of tumor-infiltrated lymphocytes (TILs) are associated with a more favorable prognosis of EC, as if a more robust immune response against tumor was activated (41). A substantial TIL infiltrate, with a high CD8^+^/FOXP3^+^ ratio, has been indicated as a possible biomarker of response to ICIs also in EC (38, 41). On the contrary, infiltration of immune-suppressive elements in the tumor microenvironment (TME)—such as tumor-associated macrophages (TAMs)—correlates with advanced stages, higher aggressivity, and shorter survival (42). Among the other potential biomarkers, it has been evidenced that cyclooxygenase-2 (COX-2) is inversely correlated with CD8^+^ infiltration, playing a potential predictive role for ICIs. It is known that COX-2 expression relates to EC development and aggressiveness, playing a negative prognostic role (43–45). *Homo sapiens* AT-rich interactive domain 1A (ARID1A) mutations have been correlated with higher infiltrations of CD8^+^ and CD4^+^ T-cells, B cells, neutrophils, macrophages, and dendritic cells (DCs), representing potential predictive biomarkers for ICI efficacy (46).

The most recent TCGA classification could represent a starting point for better understanding the genomic and immunological features of EC in order to guide the best treatment selection: POLE-ultra-mutated tumors represent 8%–10% of endometrioid subtype and are characterized by mutation of a catalytic subunit of epsilon DNA polymerase; MSI-H tumors have high mutation rate and are found in sporadic and inherited EC; copy number-low included the majority of endometrioid subtype, having a low mutation rate and frequent mutations of phosphatase and tensin homolog (PTEN), phosphoinositide 3-kinase (PI3KCA), ARID1A, Kirsten rat sarcoma virus (KRAS), and catenin beta-1 (CTNNB1) genes; copy number-high included serous and 25% of endometrioid tumors, having a high copy number variation but low mutation rate, TP53 mutations, low hormone receptor expression, very similar to triple-negative breast cancer, and serous ovarian cancer (11). The classification of TCGA is intriguing, as POLE-mutant and MSI-H EC correspond to specific phenotypes with signs of immune activation, such as high TMB, PD1/PD-L1 overexpression, and high CD3^+^ and CD8^+^ TIL infiltrates (38, 41). Some cases are described of good response in POLE-mutant or MSI-H EC, also in histologic subtypes different from endometrioid, such as clear cells or serous—for which evidence is far more limited (47). Even if MSI seems an effective predictive marker for guiding patients’ selection so far, further investigation is needed. As emerged from the pilot NCT02899793 study, defects of MMR genes leading to MSI could differ from each other: in the study, germline mutations were associated with a meaningfully higher response to pembrolizumab than sporadic mutations. Moreover, Lynch-like versus sporadic MSI, as well as the different genetic alterations, also has a prognostic significance (22, 48). Effectively, whether mechanisms underlying MSI characterize ICI sensitivity is unclear, and pathways leading to ICI resistance remain unknown. Therefore, future studies should evaluate ICIs and their combinations in different subtypes of MSI patients but also resistance mechanisms to ICIs and treatment after progression. Combination of ICIs with drugs having a different mechanism of action could be helpful to overcome ICI resistance, as preliminary results of the exploratory cohort of the NCT03367741 trial show: among the 20 immunotherapy-progressive women, 6 responses and 8 SD to nivolumab plus cabozantinib were recorded (28). Far less is known about the role of POLE mutation for ICI response, which should be further investigated. A single patient reaching an SD after pembrolizumab was reported in the KEYNOTE-028 trial, and other good responses to ICIs are described, but with limited data (44, 45). Effectively, nivolumab induced an ORR of 50% in patients with pathogenic POLE mutations and MMRp treated with nivolumab in the exonucleasic domain-mutated (ed) POLE cohort of the phase II NCT03012581 trial, of which 4/16 were diagnosed with EC (46–49). The integration of molecular and immune classification could be helpful to guide best patients’ selection.

Regarding MSS EC, the combination of ICIs and TKIs seems effective. Multikinase TKIs have been associated with a decrease in immunosuppressive elements such as TAMs and increase in CD8^+^ T cells, inducing immune activation, and they upregulate PD-L1 and Tregs that, in turn, promote angiogenesis (50–52). However, after these studies, concerns about the safety profile have emerged, as 2 out of 3 patients developed serious adverse events (24–26, 28). Like other tumor subtypes, combination treatments represent future options for EC, and currently, studies are focusing on the association with other drugs. Many trials are ongoing, most frequently regarding the combination of ICIs and chemotherapy, radiotherapy, PARP inhibitors, and tyrosine-kinase inhibitors; also, some studies are targeting the adjuvant setting. Effectively, chemotherapy holds immunomodulant properties: for example, platinum compounds can upregulate the class I major histocompatibility complex (MHC), recruit effector T cells and stimulate their cytotoxicity, and downregulate immunosuppressive elements of the TME (53). Antiangiogenics directly influence TME, increasing TILs, favoring dendritic cell maturations and T-cell infiltration (54). PARP inhibitors increase CD4^+^ and CD8^+^ T-cells, class II MHC, and immune mediators such as PD1, interferon (IFN) gamma and tumor necrosis factor (TNF) alpha, decreasing inhibitory elements such as T-cell immunoglobulin domain and mucin domain 3 (Tim-3), lymphocyte-activation gene 3 (LAG-3), and PD1 (55). Finally, other immunomodulant pathways such as LAG-3, indoleamine 2,3-dioxygenase (IDO), and Janus kinase (JAK) represent complementary axes for improving immune response and potentiate anti-PD/PD-L1 (**Table 2**). Another potential combination is with radiotherapy, which indeed holds a central role for treating EC both with curative intent in the localized stage and as symptom palliation in the metastatic setting (7). In fact, radiation causes cancer cell damage, exposing tumor antigens and activating immune response after priming T cells. Moreover, radiotherapy modulates TME, favoring the infiltration of immune cells at tumor sites (56). Therefore, the combination of ICIs and RT is under evaluation, especially in the localized setting (**Table 2**). All the studies we included in our systematic review have been conducted in pretreated patients. Nonetheless, it could be of interest to evaluate if an earlier ICI start is feasible and effective for advanced EC patients, for planning a correct sequence strategy. Currently, studies of ICIs and chemotherapy or PARP inhibitors combinations are ongoing in naïve patients (**Table 2**).

**Table 2:** Ongoing trials of ICI combinations in EC.

clinicaltrials.gov registration	Phase	Setting	ICIs	Combination (drug class)
*Advanced/recurrent EC*				
NCT03276013 (TOPIC)	II	Pretreated EC	Pembrolizumab	Doxorubicin
NCT03914612	III	Untreated EC	Pembrolizumab	Paclitaxel, carboplatin
NCT03835819	II	Pretreated MSS FRalpha+ EC	Pembrolizumab	Mirvetuximab soravtansine/IMGN853 (ADC)
NCT02549209	II	Untreated or platinum-sensitive EC	Pembrolizumab	Carboplatin, paclitaxel
NCT04014530 (ATAPEMBRO)	I-II	Pretreated MMRd EC	Pembrolizumab	Ataluren (anti non-sense mutations of DNA)
NCT05036681	II	Untreated or pre-treated MSS EC	Pembrolizumab	Futibatinib (anti-FGFR)
NCT04652076 (GYNET)	I-II	Pretreated EC	Pembrolizumab	Carboplatin, paclitaxel, NP-137 (anti-Netrin1)
NCT04865289 (ENGOT-en9/MK-7902-001, LEAP-001)	III	Untreated EC	Pembrolizumab	Carboplatin, paclitaxel, lenvatinib (TKI)
NCT04781088	II	Pretreated EC	Pembrolizumab	Paclitaxel, lenvatinib (TKI)
NCT02646748	I	Pretreated EC	Pembrolizumab	Itacitinib/INCB050465 (JAK inhibitor)
NCT03454451	I	Pretreated EC	Pembrolizumab	Ciforadenant/CPI-006 (anti-CD73 antibody)
NCT05039801	I	Pretreated EC	Pembrolizumab	IPN60090 (glutaminase inhibitor)
NCT03849469	I	Pretreated EC	Pembrolizumab	XmAb22841 (bi-specific anti-CTLA4/anti-LAG3 antibody)
NCT04278144	I-II	HER-2^+^ pre-treated EC	Pembrolizumab	BDC-1001 (anti-HER2)
NCT04460456	I	HER-2^+^ pre-treated EC	Pembrolizumab	SBT6050 (anti-HER2)
NCT03367741	II	Pretreated EC	Nivolumab	Cabozantinib (TKI)
NCT04106414	II	Pretreated EC	Nivolumab	BMS-986205 (IDO inhibitor)
NCT04423029	I-II	Pretreated EC	Nivolumab	DF6002 (anti-IL12 receptor)
NCT03667716	I	Pretreated EC	Nivolumab	COM701 (PVRIG inhibitor)
NCT03508570	I	Pretreated EC	Nivolumab, Ipilimumab	Double ICIs (anti-PD1/anti-CTLA4)
NCT04570839	I-II	Pretreated EC	Nivolumab	COM701 (PVRIG inhibitor), BMS-986207 (anti-TIGIT)
NCT04042116	I-II	Pretreated EC	Nivolumab	Lucitanib (anti-VEGFR1-3)
NCT03126110	I-II	Pretreated EC	Nivolumab, Ipilimumab	INCAGN01876 (anti-GITR)
NCT02912572	II	Pretreated EC	Avelumab	Talazoparib (PARP inhibitor), axitinib (TKI)
NCT03503786 (MITO END-3)	II	Pretreated EC	Avelumab	Carboplatin, paclitaxel
NCT03603184 (AtTEnd)	III	Untreated EC	Avelumab	Carboplatin, paclitaxel
NCT03526432	II	Pretreated EC	Atezolizumab	Bevacizumab (anti-VEGF)
NCT04486352	I-II	Pretreated EC	Atezolizumab	Bevacizumab (anti-VEGF), ipatasertib (AKT inhibitor), talazoparib (PARP inhibitor)
NCT03694262 (EndoBARR)	II	Pretreated EC	Atezolizumab	Rucaparib (PARP inhibitor), bevacizumab (anti-VEGF)
NCT03170960	I-II	Pretreated EC	Atezolizumab	Cabozantinib (TKI)
NCT04269200	III	Untreated EC	Durvalumab	Carboplatin, paclitaxel, olaparib (PARP inhibitor) maintenance
NCT04444193	NA	Untreated EC	Durvalumab	Lenvatinib (TKI)
NCT03951415 (DOMEC)	II	Untreated or pretreated EC	Durvalumab	Olaparib (PARP inhibitor)
NCT03660826	II	Pretreated EC	Durvalumab	Capivasertib (AKT inhibitor), cediranib (anti-VEGFR), olaparib (PARP inhibitor)
NCT03277482	I	Pretreated EC	Durvalumab, Tremelimumab	RT
NCT03983954	I	Pretreated EC	Durvalumab	Obinutuzumab (anti-CD20), naptumomab estafenatox (anti-5T4)
NCT03981796	III	Untreated EC	Dostarlimab	Carboplatin, paclitaxel-niraparib
*Adjuvant EC*				
NCT03694834	I	Neoadjuvant/adjuvant EC	Pembrolizumab	Single dose before surgery, then combined with adjuvant CT
NCT03932409 (FIERCE)	I	Neoadjuvant/adjuvant EC	Pembrolizumab	Single dose before RT (brachytherapy), then combined with adjuvant CT
NCT04214067	III	Adjuvant EC	Pembrolizumab	Plus RT vs. RT alone, stage II/III MSI
NCT04634877 (Keynote-B21)	III	Adjuvant EC	Pembrolizumab	Added to adjuvant CT +/- RT

ADC, antibody–drug conjugate; AKT, AK strain transforming; CD, cluster of differentiation; CT, chemotherapy; FGFR, fibroblast growth factor receptor; FR, folate receptor; GITR, glucocorticoid-induced tumor necrosis factor receptor; HER-2, human epidermal growth factor receptor 2; IDO, indoleamine 2,3-dioxygenase; IL, interleukin; JAK, Janus kinase; LAG-3, lymphocyte-activation gene 3; MMRd, mismatch-repair deficient; MSS, microsatellite stable; NA, not applicable; PARP, poly(ADP-ribose) polymerase; PVRIG, poliovirus receptor-related immunoglobulin domain containing; RT, radiation therapy; TIGIT, T-cell immunoreceptor with Ig and ITIM domains; TKI, tyrosine-kinase inhibitor; VEGF, vascular endothelial growth factor; VEGFR, VEGF-receptor.

Our analysis has several potential limitations. First is the heterogeneity of the included trials, in terms of treated patients, biomarker selection, and endpoints. We did not conduct a quantitative comparative meta-analysis due to the non-comparative design of the almost totality of included trials, and therefore the conclusions drawn about the efficacy and safety of ICIs in EC from our work are only descriptive. Moreover, OS data are incomplete: a longer follow-up is needed to clarify the real impact on survival of ICIs for EC patients. Furthermore, in many studies, safety data are partially reported. Data from randomized trials comparing ICIs with other treatments are warranted to validate efficacy and safety outcomes.

## 5 Conclusions

The results of our systematic review demonstrate that ICIs are effective and well-tolerated in patients with pretreated advanced/recurrent EC. To the best of our knowledge, it is the first systematic review focusing on this topic. With single agents, the highest responses are observed among MSI patients. MSS patients benefit more from the combination of pembrolizumab and lenvatinib, notwithstanding with worse toxicity than ICIs alone. So far, no advantages have derived from the double PD1/CTLA4 blocking. Randomized clinical trials are expected. Given the exciting results in MSI-H patients, MMR status should be investigated in every advanced EC patient at diagnosis. On the contrary, PD-L1 as a unique biomarker cannot predict ICI response in EC. For sure, accurate predictive biomarkers are warranted, as well as further studies investigating resistance mechanisms and treatment after ICI progression. So far, clinical trials have focused on pretreated patients, but the impact of ICIs both as single agents and as combinations should be investigated in naïve patients.

## Data Availability Statement

The original contributions presented in the study are included in the article/**Supplementary Material**. Further inquiries can be directed to the corresponding author.

## Author Contributions

BM: conceptualization, methodology, formal analysis, software, investigation, data curation, original draft preparation, review, editing. MM: software, investigation, data curation, original draft preparation. GC: validation, supervision, visualization. AM: visualization. DL: manuscript review, validation, supervision, visualization. EM: editing, validation, supervision, visualization. All authors contributed to the article and approved the submitted version.

## Conflict of Interest

The authors declare that the research was conducted in the absence of any commercial or financial relationships that could be construed as a potential conflict of interest.

## Publisher’s Note

All claims expressed in this article are solely those of the authors and do not necessarily represent those of their affiliated organizations, or those of the publisher, the editors and the reviewers. Any product that may be evaluated in this article, or claim that may be made by its manufacturer, is not guaranteed or endorsed by the publisher.
