# Supplementary material for: How Immunotherapy Modified the Therapeutic Scenario of Endometrial Cancer: A Systematic Review
Source: Front Oncol. 2022 Apr 14;12:844801. doi: 10.3389/fonc.2022.844801 (PMC9047829; doi:10.3389/fonc.2022.844801)
Supplement: Supplementary file 1 [file DataSheet_1.docx]

|  | **Confounding bias** | **Selection bias** | **Classification intervention bias** | **Deviation from intended intervention** | **Missing data bias** | **Measure outcome bias** | **Selected outcome bias** | **Overall bias** |
| --- | --- | --- | --- | --- | --- | --- | --- | --- |
| Marabelle et al (NCT02628067) | 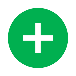 | 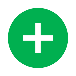 | 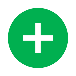 | 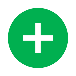 | 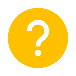 | 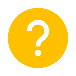 | 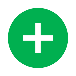 | 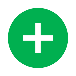 |
| Le et al  (NCT01876511) | 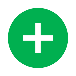 | 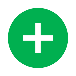 | 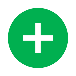 | 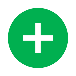 | 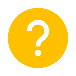 | 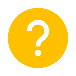 | 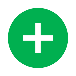 | 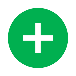 |
| Ott et al (NCT02054806) | 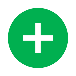 | 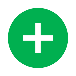 | 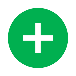 | 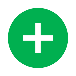 | 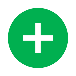 | 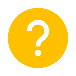 | 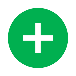 | 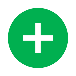 |
| Fader et al | 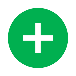 | 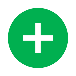 | 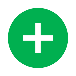 | 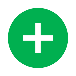 | 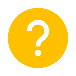 | 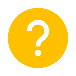 | 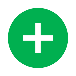 | 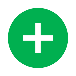 |
| Roque et al  (NCT02909793) | 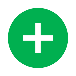 | 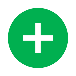 | 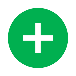 | 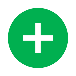 | 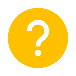 | 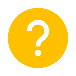 | 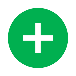 | 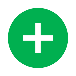 |
| Makker et al (NCT02501096) | 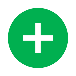 | 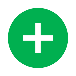 | 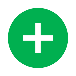 | 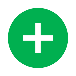 | 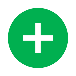 | 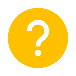 | 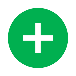 | 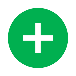 |
| Makker et al (NCT03517449) | 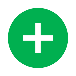 | 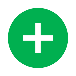 | 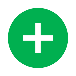 | 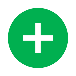 | 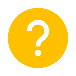 | 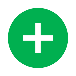 | 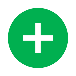 | 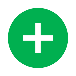 |
| Taylor et al  (NCT02501096) | 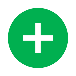 | 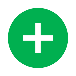 | 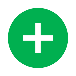 | 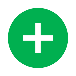 | 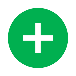 | 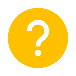 | 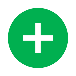 | 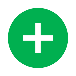 |
| Tamura et al  (JapicCTI-163212) | 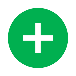 | 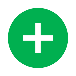 | 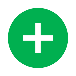 | 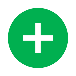 | 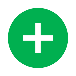 | 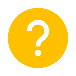 | 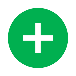 | 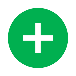 |
| Lheureux et al  (NCT03367741) | 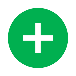 | 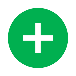 | 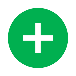 | 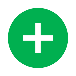 | 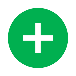 | 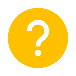 | 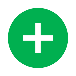 | 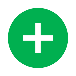 |
| Oaknin et al (NCT02715284) | 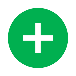 | 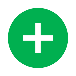 | 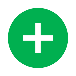 | 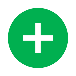 | 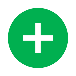 | 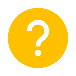 | 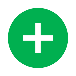 | 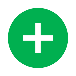 |
| Konstantinopoulos et al (NCT02912572) | 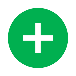 | 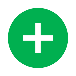 | 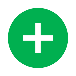 | 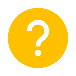 | 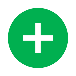 | 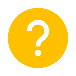 | 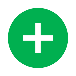 | 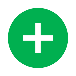 |
| Fleming et al  (NCT01375842) | 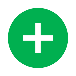 | 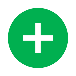 | 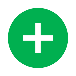 | 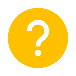 |  |  |  |  |
| Antill et al  (ANZGOG1601) |  |  |  |  |  |  |  |  |
| Rubinstein et al  (NCT03015129) |  |  |  |  |  |  |  |  |

**Supplementary Figure 1. ROBINS-I tool for risk of bias.**
